# Identification of cell-biologic mechanisms of coronary artery spasm and its ex vivo diagnosis using peripheral blood-derived iPSCs

**DOI:** 10.1186/s40824-023-00345-2

**Published:** 2023-02-18

**Authors:** Han-Mo Yang, Joo-Eun Lee, Ju-Young Kim, Jihye You, Joonoh Kim, Hak Seung Lee, Hee Min Yoo, Min Gyu Kong, Jung-Kyu Han, Hyun-Jai Cho, Kyung Woo Park, Hyun-Jae Kang, Bon-Kwon Koo, Young-Bae Park, Hyo-Soo Kim

**Affiliations:** 1 Department of Internal Medicine, Seoul National University Hospital, 101 Daekak-Ro, Chongno-Gu, Seoul, 03080 Korea; 2 National Research Laboratory for Stem Cell Niche, Seoul, Korea; 3 Innovative Research Institute for Cell Therapy, Seoul National University Hospital, Seoul, Korea; 4 Biometrology Group, Korea Research Institute of Standards and Science (KRISS), Daejeon, Korea; 5 Department of Internal Medicine, Soon Chun Hyang University Hospital, Bucheon, Korea; 6 Molecular Medicine and Biopharmaceutical Sciences, Seoul National University, Seoul, 03080 Korea

**Keywords:** Coronary artery spasm, Vasospastic angina, Calcium, Ex vivo diagnosis

## Abstract

**Background:**

Although vasospastic angina (VSA) is known to be caused by coronary artery spasm, no study has fully elucidated the exact underlying mechanism. Moreover, in order to confirm VSA, patients should undergo invasive coronary angiography with spasm provocation test. Herein, we investigated the pathophysiology of VSA using peripheral blood-derived induced pluripotent stem cells (iPSCs) and developed an ex vivo diagnostic method for VSA.

**Methods and results:**

With 10 mL of peripheral blood from patients with VSA, we generated iPSCs and differentiated these iPSCs into target cells. As compared with vascular smooth muscle cells (VSMCs) differentiated from iPSCs of normal subjects with negative provocation test, VSA patient-specific iPSCs-derived VSMCs showed very strong contraction in response to stimulants. Moreover, VSA patient-specific VSMCs exhibited a significant increase in stimulation-induced intracellular calcium efflux (Changes in the relative fluorescence unit [ΔF/F]; Control group vs. VSA group, 2.89 ± 0.34 vs. 10.32 ± 0.51, *p* < 0.01), and exclusively induced a secondary or tertiary peak of calcium efflux, suggesting that those findings could be diagnostic cut-off values for VSA. The observed hyperreactivity of VSA patient-specific VSMCs were caused by the upregulation of sarco/endoplasmic reticulum Ca^2+^-ATPase 2a (SERCA2a) due to its enhanced small ubiquitin-related modifier** (**SUMO)ylation. This increased activity of SERCA2a was reversed by treatment with ginkgolic acid, an inhibitor of SUMOylated E1 molecules (pi/µg protein; VSA group vs. VSA + ginkgolic acid, 52.36 ± 0.71 vs. 31.93 ± 1.13, *p* < 0.01).

**Conclusions:**

Our findings showed that abnormal calcium handling in sarco/endoplasmic reticulum could be induced by the enhanced SERCA2a activity in patients with VSA, leading to spasm. Such novel mechanisms of coronary artery spasm could be useful for drug development and diagnosis of VSA.

**Supplementary Information:**

The online version contains supplementary material available at 10.1186/s40824-023-00345-2.

## Background

Coronary artery spasm plays an important role in vasospastic angina (VSA) and can manifest through diverse clinical features, including angina pectoris, acute coronary syndrome, and sudden cardiac death [1–5]. Coronary artery spasm can occur not only in normal arteries, but also in fixed atherosclerotic lesions [6]. Moreover, it can be induced by exercises or stress conditions [6]. Since all of these characteristics make a diagnosis of coronary artery spasm very difficult, overlooked spasm in patients with VSA could result in fatal complications such as ST-segment elevation myocardial infarction or fatal ventricular arrhythmia, which lead to sudden cardiac death [3, 7–10]. In order to prevent such fatal outcomes, pharmacological treatments as well as cardioverter defibrillator implantation have been attempted [11].

Until now, vascular smooth muscle cells (VSMCs) hypersensitivity or endothelial cells (ECs) dysfunction are known possible mechanism for coronary artery spasm [12, 13]. However, there have been no studies to elucidate the exact underlying mechanism of coronary artery spasm in terms of cell-biologic and molecular aspect. Moreover, in order to confirm VSA, patients should undergo invasive spasm provocation test during coronary angiography. From 10 mL of peripheral blood, we recently isolated and cultured human endocardium-derived circulating multipotent stem (CiMS) cells, which could be easily reprogrammed and differentiated into target tissue cells including vascular cells [14]. Using this method, we aimed to develop more simple and practical diagnostic method. Here, we investigated the biologic mechanism of VSA using peripheral blood-derived induced pluripotent stem cells (iPSCs) and developed an ex vivo diagnostic method for VSA.

## Materials and methods

All human samples were obtained with written informed consent after the approval by the Institutional Review Board (IRB) of Seoul National University Hospital (H-0908–036-290). All animal experiments were performed after receiving approval from the Institutional Animal Care and Use Committee (IACUC) of Clinical Research Institute in Seoul National University Hospital and complied with the National Research Council (NRC) ‘Guidelines for the Care and Use of Laboratory Animals’ (SNU-100106–2). The characteristics of subjects are summarized in Supplemental Table S1 (normal control *n* = 13; vasospastic angina *n* = 15). Coronary angiography of all subjects included in our study showed insignificant coronary stenosis. During coronary angiography with a provocation test using ergonovine, we checked all of the following in response to the stimulus: 1) > 90% vasoconstriction on coronary angiography 2) reproduction of the usual chest pain 3) ischemic ECG change. Peripheral blood samples were obtained from VSA patients before angiography.

### Peripheral blood mononuclear cells (PBMNCs) isolation and circulating multipotent stem (CiMS) cell cultures

Human peripheral blood samples (10 mL) were obtained from blood donors with informed consent. PBMNCs were isolated from the blood samples via density gradient centrifugation with Ficoll-Paque PLUS (GE Healthcare, Piscataway, NJ). The blood samples were diluted with 20 mL room temperature phosphate-buffered saline (PBS) and gently inverted several times. A 30 mL layer of diluted human peripheral blood was carefully formed above 12 mL of Ficoll-Paque PLUS (GE Healthcare, Piscataway, NJ) in a 50 mL conical tube. This was followed by centrifugation at 2500 rpm for 30 min at 20 °C without any brakes. The upper layer was subjected to aspiration, and the mononuclear cell layer was carefully transferred to a new 50 mL conical tube. The tube was filled with cold PBS, gently mixed, then centrifuged at 1800 rpm for 10 min at 4 °C. The supernatant was thoroughly removed and the cell pellet was washed with cold PBS by centrifuging at 1800 rpm for 10 min at 4 °C.

Freshly isolated mononuclear cells were suspended with the EGM-2MV BulletKit system (Lonza, Basel), which contains endothelial basal medium-2, 5% fetal bovine serum (FBS), human epidermal growth factor (hEGF), vascular endothelial growth factor (VEGF), human fibroblast growth factor-basic (hFGF-B), insulin growth factor-1 (R3-IGF-1), and ascorbic acid. Mononuclear cells were seeded on non-coated six-well plates (Sigma, St. Louis, MO) at 6 × 10^6^ cells/6-well and incubated in a 5% CO_2_ incubator at 37 °C. CiMS cells were cultured as described in a previous study [14]: in brief, the medium was changed daily for up to 2 weeks after plating. Adherent CiMS cells were observed as early as 3 days after the start of the culture, gradually forming colonies. CiMS cell colonies grown in the culture were maintained with EGM-2MV media and sub-cultured using 0.05% Trypsin–EDTA solution (Invitrogen, Carlsbad, CA). CiMS cells were passaged every 3 to 4 days.

### Retro virus infection and iPSCs generation

Human embryonic kidney (HEK) 293 T cells were plated at 1 × 10^6^ cells/100 mm-dish and incubated overnight. In the following day, the cells were transfected with pMXs vectors using polyethylenimine (Polyscience, Warrington, PA) as a transfection reagent. 48 h after transfection, the medium was collected as the first virus-containing supernatant and replaced with a fresh medium, which was collected after 24 h as the second virus-containing supernatant. Normal- and VSA- CiMS cells were seeded at 5 × 10^5^ cells/6-well 1 day before transduction. The virus-containing supernatants were filtered through a 0.22 mm pore-size filter and concentrated using ultrahigh centrifugation at 25,000 rpm for 90 min. Viral supernatant was supplemented with 10 μg/mL polybrene (Sigma-Aldrich, St. Louis, MO). Equal amounts of supernatants containing each of the four retroviruses were mixed, transferred to the CiMS cell dish, and incubated overnight. 24 h after transduction, the virus-containing medium was replaced with EGM-2MV medium (Lonza, Basel). 6 days after transduction, CiMS cells were harvested by trypsinization and re-plated at 2.5 × 10^5^ cells/35 mm dish on an STO feeder cell layer. 2 days later, the medium was replaced with Primate ES Cell Medium supplemented with 10 ng/mL basic fibroblast growth factor (bFGF). The medium was changed every other day. 14 days after transduction, colonies were picked up and transferred into 2 mL of Primate embryonic stem (ES) cell medium. The colonies were mechanically dissociated into small clumps via pipetting. The cells were transferred on STO in 6-well plates.

### Intracellular calcium measurement

Normal control- and VSA patient-derived VSMCs were cultured and dissociated in 0.25% Trypsin–EDTA solution (Invitrogen, Carlsbad, CA). The VSMCs were seeded at a concentration of 5 × 10^4^ cells/well on collagen type IV (Sigma-Aldrich, St. Louis, MO)-coated 35 mm-confocal dishes (ibidi, Gräfelfing) in human VSMCs differentiation media and incubated at 37 °C. The cells were loaded with 1 μM Fluo-4 (Invitrogen, Carlsbad, CA) in PBS containing 1% FBS (Invitrogen, Carlsbad, CA), and 2.5 mM probenecid (Sigma-Aldrich, St. Louis, MO). Cells were incubated with Fluo-4 dye mixture at 37 °C for 15 min, which was then left for 15 min at room temperature to allow for the de-esterification of the indicator. Cells were washed with PBS and replaced with 1.5 mL PBS containing 1% FBS solution. Cells were then treated with vasoactive agonists, such as carbachol (250 μM, Sigma-Aldrich, St. Louis, MO), ergonovine (250 μM, Tocris Bioscience, Bristol), and acetylcholine (250 μM, Sigma-Aldrich, St. Louis, MO), as well as vasodilators and calcium channel blockers, such as Verapamil (250 μM, Sigma-Aldrich, St. Louis, MO), Diltiazem (250 μM, Sigma-Aldrich, St. Louis, MO), Nicorandil (250 μM, Sigma-Aldrich, St. Louis, MO), Molsidomine (250 μM, Sigma-Aldrich, St. Louis, MO), and Nitrate (250 μM, Sigma-Aldrich, St. Louis, MO). The vasoactive agonists, vasodilators, and calcium channel blockers were manually loaded with volumes of 150 μL after an elapsed time of 20 s during a time-lapse confocal imaging examination using an A1 confocal laser microscope (Nikon, Melville, NY). Images were acquired at approximately 2-s intervals for a period of 10 min at 20 × magnification. Raw fluorescence intensity was expressed using the NIS-Elements C software (Nikon, Melville, NY) and the intensity of each cell was calculated with the expression ΔF/F. An ΔF/F indicated the change in the relative fluorescence unit, and all the peak values of ΔF/F were added to the final calculated value of ΔF/F.

### Adaptation to single cell-based non-colony-type monolayer cultures of iPSCs

Human iPSCs, initially grown as colonies on STO feeder cells, were cultured on human embryonic stem cell (ESC)-qualified Matrigel (BD Biosciences, San Jose, CA) for 5 days in an adaptation feeder free system. The cells were dissociated via incubation with Accutase (Invitrogen, Carlsbad, CA) for 15 min. Dissociated single cells were plated on human ESC-qualified Matrigel (BD Biosciences, San Jose, CA)-coated 6-well dishes. Approximately 2.5 × 10^5^ cells were seeded in each well of a 6-well plate in mTeSR™1 (Stemcell Technologies, Vancouver, BC).

### In vitro differentiation

• VSMCs differentiation: To induce VSMCs, single cell-based human iPSCs were treated with RPMI1640 (Invitrogen, Carlsbad, CA) containing B27 supplement (Invitrogen, Carlsbad, CA) and 10 μM CHIR99021 (Cayman chemical, Ann Arbor, MI) as well as 1% penicillin and streptomycin (Invitrogen, Carlsbad, CA) for 2 days to differentiate the mesendoderm lineage. After 2 days, the mesendoderm lineage cells were treated with RPMI1640 (Invitrogen, Carlsbad, CA) containing B27 supplement (Invitrogen, Carlsbad, CA) and 100 ng/mL BMP4 (R&D Systems, Minneapolis, MN) as well as 10 μM of LY 294002 (Sigma-Aldrich, St. Louis, MO) for 24 h to differentiate the mesoderm progenitor cells. For the VSMCs differentiation, the medium was replaced with RPMI1640 (Invitrogen, Carlsbad, CA) containing B27 supplement (Invitrogen, Carlsbad, CA) with 20 ng/mL platelet-derived growth factor-BB (PDGF-BB, R&D Systems, Minneapolis, MN) and 2 ng/mL transforming growth factor beta-1 (TGF-β1, PeproTech, Rocky Hill, NJ). The medium was changed daily. On day 5, the cells were harvested through dissociation with 0.25% Trypsin–EDTA solution (Invitrogen, Carlsbad, CA) and seeded at a concentration of 5 × 10^5^ cells/well on collagen type IV (Sigma-Aldrich, St. Louis, MO)-coated 6-well plates. The differentiation medium was changed for 20 days.
• ECs differentiation: To induce ECs differentiation, human iPSCs were treated with RPMI1640 (Invitrogen, Carlsbad, CA) containing B27 supplement (Invitrogen, Carlsbad, CA) and 10 μM CHIR99021 (Cayman chemical, Ann Arbor, MI) as well as 1% penicillin and streptomycin (Invitrogen, Carlsbad, CA) for 2 days to differentiate the mesendoderm lineage. After 2 days, the mesendoderm lineage cells were treated with RPMI1640 (Invitrogen, Carlsbad, CA) containing B27 supplement (Invitrogen, Carlsbad, CA) 100 ng/mL bone morphogenetic protein 4 (BMP4, R&D Systems, Minneapolis, MN), 100 ng/mL vascular endothelial growth factor A (VEGF-A, PeproTech, Rocky Hill, NJ), and 20 ng/mL bFGF (R&D Systems, Minneapolis, MN) for 8 days. Single cells were dissociated using Accutase (Invitrogen, Carlsbad, CA) and, after gentle pipetting, passed through 40 μm cell strainers (BD Biosciences, San Jose, CA). The remaining 5 × 10^5^ cells were subject to antibody binding with 100 μL, 1:5 dilution, anti-CD34 microbeads (Miltenyi Biotec, Bergisch Gladbach) for 15 min at 4 °C. The CD34 positive cells were magnetically sorted at room temperature using MS columns (Miltenyi Biotec, Bergisch Gladbach) and seeded on fibronectin (Sigma-Aldrich, St. Louis, MO)-coated dishes. The medium was changed daily.

### Functional contraction assay

Normal and VSA patient-derived VSMCs were cultured and dissociated using 0.05% Trypsin–EDTA solution (Invitrogen, Carlsbad, CA.) and seeded at a concentration of 5 × 10^4^ cells/well on collagen type IV (Sigma-Aldrich, St. Louis, MO)-coated 35 mm-confocal dishes (ibidi, Gräfelfing), which were incubated at 37 °C. After 24 h, VSMCs were gently washed with PBS. For contraction, 1 mM carbachol (Sigma-Aldrich, St. Louis, MO) was added to the VSMCs differentiation media and incubated at 37 °C for 60 min. Images were acquired using an Olympus IX71 microscope (Olympus, Tokyo) and the contracting area of the iPSC-derived vascular smooth muscle was automatically measured using the Image J software (National Institutes of Health, Bethesda, MD). To calculate the area, we drew lines on the image along the area and then measured the length of the lines using the software.

### Collagen gel contraction assay

Collagen gel contraction assays for 2-dimensional (2D) sheet model were examined using a collagen cell contraction assay kit (Cell biolabs, Inc, San Diego, CA) to compare the hyperreactivity of normal- and VSA patient-derived VSMCs. Human VSMCs were harvested and resuspended in collagen solution at 1 × 10^6^ cells/mL. Next, we added the cell-collagen mixture to individual wells in a 24-well plate and incubated the mixture at 37 °C for 60 min. After incubation, we gently released collagen gels from the sides of the dishes and added 1 mL of human VSMCs differentiation medium containing 1 mM carbachol (Sigma-Aldrich, St. Louis, MO) for 2 days. Collagen gel was acquired using an Olympus IX71 microscope (Olympus, Tokyo) and the area of the collagen gel was quantified using the Image J software (National Institutes of Health, Bethesda, MD).

### Immunofluorescent staining and confocal microscopy analysis

Cells were fixed with 4% paraformaldehyde for 10 min, permeabilized with 95% methanol (MeOH) for 5 min at -20 °C, and washed with PBS containing 0.05% Tween-20. After washing, the cells were blocked with 1% bovine serum albumin (BSA) in PBS for 1 h. The cells were incubated overnight at 4 ℃ with specific primary antibodies: anti-NANOG (1:100, Cell Signaling Technology, Danvers, MA), anti-OCT3/4 (1:100, Cell Signaling Technology, Danvers, MA), anti-Calponin1 (1:500, CNN1; Sigma-aldrich, MO), anti-smooth muscle actin (1:100, SMA; Abcam, Cambridge), anti-sarco/endoplasmic reticulum Ca^2+^-ATPase 2a (SERCA2a, 1:100, Abcam, Cambridge), anti-platelet endothelial cell adhesion molecule (PECAM, Santa Cruz Biotechnology, Dallas, TX), and anti-vascular endothelial cadherin (VECAD, Santa Cruz Biotechnology, Dallas, TX). This was followed by incubation with fluorescent-tagged secondary antibodies (1:100, Invitrogen, Carlsbad, CA). Images were acquired using a confocal laser scanning microscope system (LSM 710, Carl Zeiss AG, Oberkochen) and processed with the Zen software (Carl Zeiss AG, Oberkochen). A water- or oil-immersion objective lens (40 × , or 63 × , 1.4 numerical aperture, NA) with the pinhole set for a section thickness of 0.8 μm (pinhole set to 1 airy unit in each channel) was used.

### Human VSMCs spheroid formation

Normal- and VSA patient-derived VSMCs were seeded at 1.5 × 10^6^ cells/well in 60 mm cultured ultra-low attachment culture dishes (Sigma-Aldrich, St. Louis, MO) in 4 mL human VMSCs differentiation medium. After 24–36 h, the supernatant was discarded and the spheroids were washed once with RPMI1640 and seeded in ultra-low attachment culture dishes. The differentiation media was changed daily for 14 days.

### Alkaline phosphatase (AP) staining and karyotyping

AP staining was performed using the BCIP/NBT Substrate Kit (Agilent, Santa Clara, CA). Karyotype analysis was conducted using standard protocols for the chromosomal Giemsa (G)-banding.

### Three-germ-layer differentiation assay

Human iPSCs were differentiated into each of the three germ layers using the base medium and differentiation supplements provided in the Human Pluripotent Stem Cell Functional Identification Kit (R&D Systems, Minneapolis, MN). After differentiation, all cells were fixed with 95% MeOH for 10 min at -20 °C. Cells were washed with PBS containing 0.05% Tween-20 and blocked with 5% BSA before being incubated overnight with anti-Orthodenticle Homeobox (OTX, 1:100, R&D Systems, Minneapolis, MN), anti-Brachyury T (T, 1:100, R&D Systems, Minneapolis, MN), and anti-SRY-Box transcription factor17 (Sox17, 1:100, R&D Systems, Minneapolis, MN) at 4 °C. The following day, cells were washed and incubated with Alexa-anti-mouse 488 (1:100, Invitrogen, Carlsbad, CA), Alexa-anti-mouse 555 (1:100, Invitrogen, Carlsbad, CA), and Alexa-anti-mouse 647 (1:100, Invitrogen, Carlsbad, CA) for 2 h. The nuclei were counterstained with DAPI (1:1000, Invitrogen, Carlsbad, CA). The three-germ-layer differentiated cells were viewed under a confocal laser scanning microscope system (LSM 710, Carl Zeiss AG, Oberkochen) and processed using the Zen software (Carl Zeiss AG, Oberkochen).

### Ribonucleic acid (RNA) extraction, reverse transcription-polymerase chain reaction (RT-PCR), and real-time PCR

Total RNA was extracted using the Trizol reagent (Invitrogen, Carlsbad, CA). 1 μg of total RNA was utilized for reverse transcription reactions using the Power cDNA synthesis kit (Applied Biosystems, Waltham, MA) and Oligo dT primers, according to the manufacturer’s instructions. PCR was performed with AmpliTaqGold (Applied Biosystems, Waltham, MA). RT-PCR amplification of *SERCA2a*, phospholamban (*PLB)*, ryanodine receptors (*RYR)*, inositol trisphosphate receptors (*IP3R)*, and *ACTIN* was performed as follows: 94 °C for 12 min, followed by 30 cycles at 94 °C for 45 s, at 55–58 °C for 45 s, and 72 °C for 50 s. Amplified PCR products were analyzed via electrophoresis on 2% agarose gel. Primer sequences are shown in Supplemental table 2. For real-time PCR, the gene expressions of *NANOG*, *OCT3/4*, *T*, *CNN1*, *SMA*, *SMA22α*, *SERCA2a*, *PECAM*, and *VeCAD* were analyzed using the Applied Biosystems 7500 Real-Time PCR system (Applied Biosystems, Waltham, MA). The reaction was carried out according to the manufacturer's protocol using SYBR Green master mix (Roche, Basel). Gene expression levels were normalized to the level of Glyceraldehyde 3-phosphate dehydrogenase (*GAPDH)* or *18S* RNA. Gene expression was quantified using the 2^−ΔΔCT^ method.

### SERCA activity assay

SERCA activity was determined using the EnzChek Phosphate Assay Kit (Thermo Fischer Scientific, Waltham, MA) according to the manufacturer’s instructions. In brief, the normal and VSA patient-derived VSMCs homogenized cells and were quantified using the bicinchoninic acid (BCA) assay (Invitrogen, Carlsbad, CA). After quantification, 10 μg of each cell lysate sample was mixed with the reaction buffer and pre-incubated for 10 min at 22 °C before being treated with 1 mM adenosine triphosphate (ATP, Sigma, St. Louis, MO). The readings were made at 360 nm as a function of time for both the experimental reaction and the control reaction using a microplate reader (Molecular devices, San Jose, CA).

### Western blot analysis

We evaluated protein amounts from whole-cell lysates, which were quantified using the BCA assay (Invitrogen, Carlsbad, CA). 50 μg of protein from each of the indicated samples were loaded in wells with a 10% SDS-PAGE gel (Bio-Rad, Alfred Nobel Drive Hercules, CA). The proteins were transferred to a nitrocellulose membrane (Bio-Rad, Alfred Nobel Drive Hercules, CA) and blocked for 1 h using 5% skim milk. Membranes were washed and incubated overnight with anti-CNN1 (1:1000, Sigma-Aldrich, St. Louis, MO), anti-SMA (1:1000, Abcam, Cambridge), anti-Tubulin (1:1000, Santa Cruz Biotechnology, Dallas, TX), anti-SERCA2a (1:1000, Abcam, Cambridge), anti-total PLB (1:1000, Abcam, Cambridge), anti-phosphorylated PLB (1:1000, Abcam, Cambridge), anti-total RYR (1:1000, Abcam, Cambridge), anti-IP3R (1:1000, Abcam, Cambridge), anti-small ubiquitin like modifier1 (SUMO1, 1:1000, Cell Signaling Technology, Danvers, MA), anti-ubiquitin like modifier activating enzyme 2 (UBA2, 1:1000, Cell Signaling Technology, Danvers, MA), anti-SUMO specific peptidase 1 (SENP1, 1:1000, Cell Signaling Technology, Danvers, MA), and anti-ACTIN (1:1000, Santa Cruz Biotechnology, Dallas, TX). The next day, the membranes were washed and incubated for 2 h with either anti-rabbit horseradish peroxidase (HRP, 1:2500. Abcam, Cambridge), anti-mouse HRP (1:2500, Abcam, Cambridge), or anti-goat HRP (1:2500, Abcam, Cambridge). Once washed, the membranes were exposed to film and developed using an auto developer. Images were quantified using the Image J software (National Institutes of Health, Bethesda, MD).

### Lentiviral transduction

We purchased the lentiviral vector pLKO.1 shSERCA2a (Open Biosystems, Huntsville, AL) and the pLVX overexpression vector (Takarabio, Nojihigashi). To further investigate the effects of gene overexpression, we subcloned a construct encoding SERCA2a and SERCA2a point mutation into the pLVX lentiviral vector. We co-transfected lentiviral constructs and packaging vectors into 293 T cells with polyethylenimine (Polyscience, Warrington, PA, USA) and collected the supernatants containing lentiviral particles after 48 h. The viral supernatants were supplemented with 10 μg/mL polybrene (Sigma-Aldrich, St. Louis, MO). Equal amounts of supernatants containing each of the lentiviruses were mixed, transferred to the target cell dish, and incubated overnight.

### Acetylated-low density lipoprotein (Ac-LDL) uptake assay

Normal and VSA patient-derived ECs were seeded at a density of 2.5 × 10^5^ cells/well in 35 mm fibronectin-coated 35 mm-confocal dishes (ibidi, Gräfelfing) and incubated at 37 °C for 24 h. The LDL uptake assay was performed by incubating the ECs with 10 mg/mL of Dil-labeled Ac -LDL (Invitrogen, Carlsbad, CA) at 37 °C for 3 h. After washing twice with PBS, the medium was replaced with serum-free EGM ((Lonza, Basel). LDL uptake was viewed under a confocal laser scanning microscope system (LSM 710, Carl Zeiss AG, Oberkochen) and processed with the Zen software (Carl Zeiss AG, Oberkochen).

### Matrigel tube formation assay

In brief, 10 mL of BD Matrigel Matrix Growth Factor Reduced (BD Bioscience) was coated on each well of a 35 mm slide confocal dish (ibidi, Gräfelfing), which was incubated at 37 °C for 1 h. To assess the ability of normal and VSA patient-derived ECs to form vascular tube-like structures, 5 × 10^4^ normal and VSA patient-derived ECs were seeded onto each well of the Matrigel-coated confocal dish and incubated at 37 °C for 18 h. The formation of vascular tube-like structures was observed using an Olympus IX71 microscope (Olympus, Tokyo). To calculate the complete tube area, we drew lines on the image along the area and then measured the length of the lines using the Image J software (National Institutes of Health, Bethesda, MD).

### Nitric oxide (NO) measurement

NO production was determined using the NO Plus Detection Kit (Intron Biotechnology, Seoul) after 24 h of cell culturing. The supernatants were collected and the absorbance values between 520–560 nm were measured using a microplate reader (Molecular devices, San Jose, CA).

### Statistical analysis

All data are presented as means ± standard error of means (SEM). The differences between experimental groups were analyzed via the student’s *t*-test or Mann–Whitney test when appropriate. Statistical analysis was performed with GraphPad Prism 7 (GraphPad Software, San Diego, CA) for the analysis and *P* values of < *0.05* were considered to be statistically significant.

## Results

### Generation of iPSCs from peripheral blood and their subsequent differentiation into vascular smooth muscle cells (VSMCs) and endothelial cells (ECs)

**Fig. 1:**
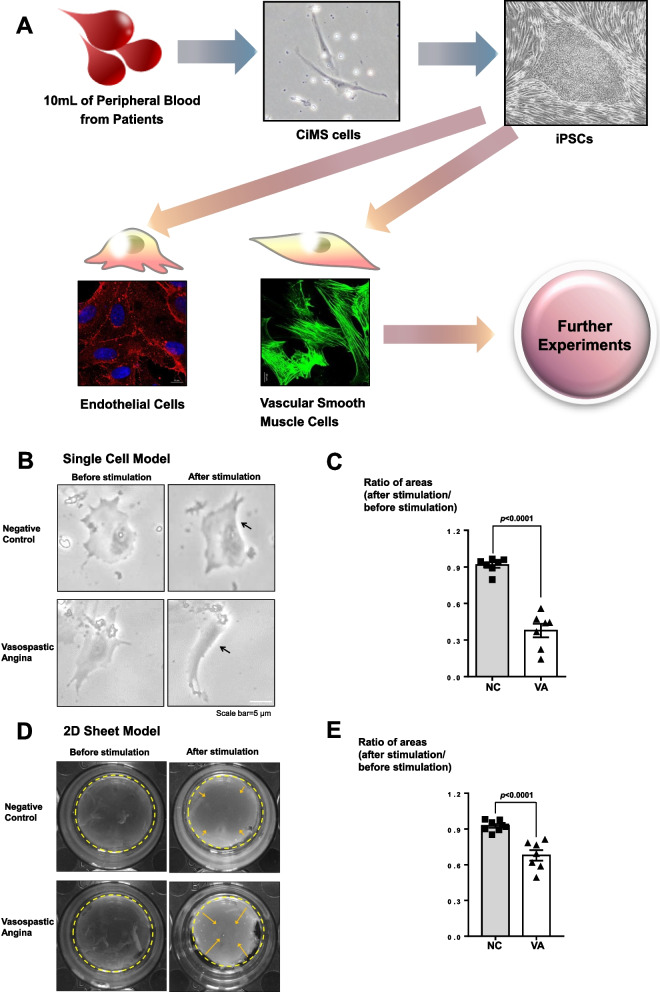
Hypercontractility of induced pluripotent stem cell (iPSC)-derived vascular smooth muscle cells (VSMCs) from vasospastic angina (VSA) patients. (**A**) Schematic summary of the study. We obtained 10 mL of peripheral blood from patients with VSA and cultured circulating multipotent stem (CiMS) cells. Thereafter, we generated VSA patient-specific iPSCs from these CiMS cells and differentiated iPSCs into both VSMCs and endothelial cells (ECs). (**B, C**) In a contraction assay using a single-cell model, 250 μM of carbachol treatment (a cholinomimetic vasoactive agonist) induced stronger contraction in VSMCs from VSA patients than in those from normal subjects. (*n* = 7 per group; scale bar, 5 μm). *NC* Negative control group with a negative provocation test, *VA* Vasospastic angina group with a positive provocation test. The black arrows denote the contraction area of the VSMCs. (**D, E**) Collagen gel contraction assays for 2 dimensional (2D) sheet models were examined using normal control subject-derived VSMCs and VSA patient-derived VSMCs. Treatment with 1 mM carbachol for 48 h induced a strong contraction of sheets derived from the VSMCs of VSA patients, as observed in the 2D sheet model (*n* = 7 per group). The areas circled with yellow dash lines denote the sizes of the collagen gels, and the yellow arrows indicate the contraction of the collagen gels

After obtaining only 10 mL of peripheral blood from VSA patients, we successfully cultured circulating multipotent stem (CiMS) cells whose cell-biologic features were fully described in our previous work [14]. Using these cells, we generated control group and VSA patient-specific iPSCs using retroviruses expressing OCT3/4, SOX2, KLF4, and c-MYC [15]. We then stained positively for alkaline phosphatase (AP) activity, and expressed the pluripotency markers such as NANOG and OCT3/4, as determined by immunocytochemistry and gene expression levels (Supplemental Fig. 1A-D). We verified three germ layer markers: ectoderm (Orthodenticle Homeobox: OTX), mesoderm (Brachyury T: T), and endoderm (SRY-Box Transcription Factor 17: SOX17) (Supplemental Fig. 1E). We also confirmed normal karyotypes and lack of tumorigenicity (Supplemental Fig. 1F-G). In VSA patient VSMCs, hyperreactivity is persistent and ECs dysfunction causes vasoconstriction [13, 16]. For this reason, we differentiated normal- and VSA patient-specific iPSCs into VSMCs and ECs (Fig. 1A, Supplemental Fig. 2A, 3A). To differentiate mesoderm lineage, we treated with GSK inhibitor (CHIR99021) and confirmed mesodermal progenitor markers such as T (Supplemental Fig. 2B-2D). Furthermore, we observed VSMCs morphology and confirmed the expression level of VSMCs markers, such as calponin1 (CNN1), smooth muscle protein 22 alpha (SMA22α), and smooth muscle actin (SMA) that showed high expression with no difference between NC- and VSA- derived VSMCs (Supplemental Fig.  2E-2I). Moreover, we differentiated to ECs and examined the expression levels of general ECs markers, such as platelet endothelial cell adhesion molecule (PECAM) and vascular endothelial cadherin (VeCAD) (Supplemental Fig. 3B-3D). Next, we investigated the ability of ECs to form tube-like structures and analyzed the uptake of Dil-labeled Acetylated-LDL (Supplemental Fig. 3E-3F). Moreover, we examined nitric oxide (NO), and found no difference between NC- and VSA- derived ECs (Supplemental Fig. 3G-3H).

**Fig. 2:**
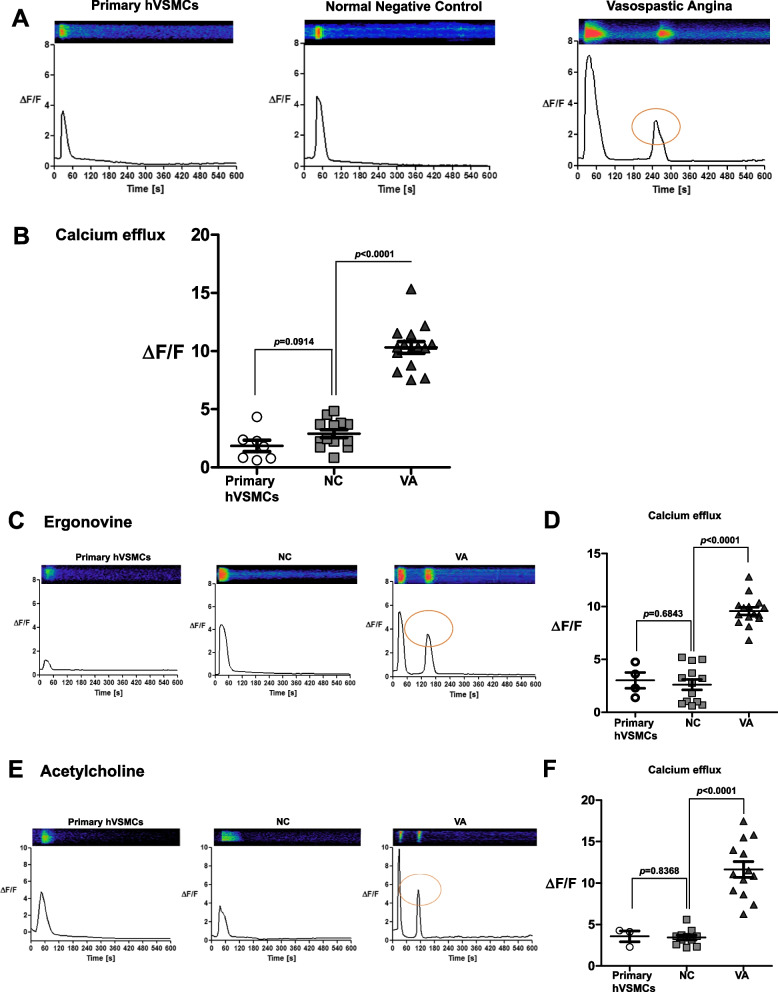
Hyperreactivity of iPSC-derived VSMCs from VSA patients was triggered by an abnormal increase of intracellular calcium efflux (**A, B**) Intracellular calcium efflux in response to carbachol was measured with Fluo-4 (a calcium fluorescent dye. Fluo-4 treated cells were monitored through time-lapse confocal microscopy over 10 min after the addition of 250 μM of carbachol, an inducer of contraction. The iPSC-derived VSMCs from VSA patients exhibited much higher intensity of the calcium efflux peak than the cells from control subjects. Moreover, secondary or tertiary peaks of calcium efflux were observed only in the VSA group (primary human VSMCs *n* = 7; NC *n* = 13; VA *n* = 15). Primary human VSMCs indicate commercially available human coronary artery vascular smooth muscle cells. The orange circle indicates the secondary peak. (**C-F**) The addition of 250 μM ergonovine and 250 μM acetylcholine in primary human VSMCs, NC- and VSA- iPSC-derived VSMCs produced similar results to treated carbachol (Ergonovine; primary human VSMCs *n* = 4; NC *n* = 13; VA *n* = 15) (Acetylcholine; primary human VSMCs *n* = 3; NC *n* = 11; VA *n* = 13). The orange circle indicates the secondary peak

**Fig. 3:**
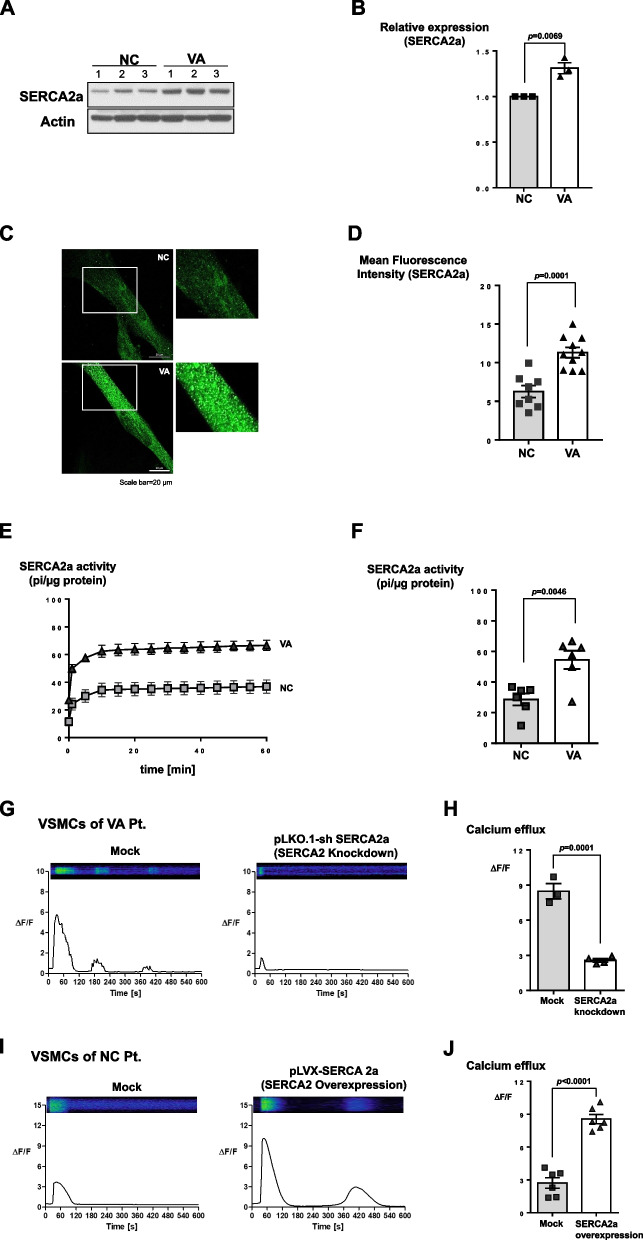
The mechanism of hyperreactivity of iPSC-derived VSMCs from VSA patient: the enhanced sarco/endoplasmic reticulum Ca^2+^-ATPase 2a (SERCA2a) activity. (**A, B**) 10 μg of whole protein lysates from each of the samples were loaded with a 10% SDS-PAGE gel, transferred to nitrocellulose membranes, and probed with anti-SERCA2a (1:1000) and anti-sarcomeric actin antibodies (1:1000). Western blot indicates that the amount of endogenous SERCA2a protein was greater in iPSC-derived VSMCs from VSA patients than from normal subjects (*n* = 3 per group). All samples in Western blot were obtained from individual subjects (three different normal subjects and three different VSA patients). (**C, D**) Immunofluorescence staining for SERCA2a shows higher intensity in iPSC-derived VSMCs from VSA patients than from normal subjects (*n *= 8–10 per group; scale bar, 20 μm). Each group of cells was stained with human anti-SERCA2a (1:100). SERCA2a was detected using the Alexa Fluor 488-conjugated anti-mouse secondary antibody (1:100). (**E, F**) Calcium-dependent ATPase activity assay indicates that the activity of SERCA2a in the VSA group was greater than in the control group (*n* = 6 per group). 10 μg of each cell lysate was mixed with the reaction buffer and pre-incubated for 10 min before being treated with 1 mM adenosine triphosphate (ATP). The ATPase activity was measured at 360 nm using a microplate reader. (G, H) After knocking down SERCA2a expression in iPSC-derived VSMCs from VSA patients using a lentiviral vector and induced with 250 μM carbachol, intracellular calcium efflux was downregulated to the levels of control cells (Mock *n* = 3; shSERCA2a *n* = 4). Mock indicates control lentiviral vector transfection. (I, J) In contrast, the overexpression of SERCA2a in iPSC-derived VSMCs from normal subjects using a lentiviral vector induced with 250 μM carbachol increased the level of intracellular calcium efflux and generated the second peak of calcium efflux (*n* = 6 per group)

### Hyperreactivity of VSMCs derived from VSA patients

We compared the VSMCs of VSA patients showing coronary spasm during provocation tests (*n* = 15) to those of subjects with negative provocation test results (*n* = 13) after confirming insignificant coronary stenosis in their coronary angiography. First, we analyzed their capacity to contract in response to treatment with carbachol, a vasoactive agonist. VSA patient-derived VSMCs exhibited very strong contraction compared to VSMCs from control subjects (Fig. 1B and 1C). We observed similar results in the 2D sheet model (Fig. 1D and 1E). Among the underlying mechanisms for VSMCs contraction, changes in intracellular calcium are considered to be the most important final pathway [17, 18]. Thus, we measured the intracellular calcium efflux and calcium sparks of VSMCs using laser-scanning confocal microscopy with Fluo-4, a calcium-indicator dye [19]. Interestingly, in response to stimulation, VSMCs from VSA patients exhibited much higher intensity of calcium efflux peaks than VSMCs from normal subjects did (Fig. 2A, Supplemental Fig. 4, and Supplemental movie 1). Moreover, a secondary or tertiary peak of calcium efflux was noted only in the VSA group. We observed a clear distinction between VSA and control group around the ΔF/F value (change in the relative fluorescence unit) of 6 or 7 (Fig. 2B). Similar results were noted in response to other stimulants such as ergonovine and acetylcholine (Fig. 2C-2F). When we analyzed intracellular calcium activity in each cell group using the 3D spheroid model, continuous and multiple sparks of intracellular calcium were observed in the VSA group (Supplemental Fig. 5A-5C, Supplemental movie 2). These data indicate that the induced hyperreactivity of the VSA group VSMCs induced may be caused by the aberrant calcium signal.

**Fig. 4:**
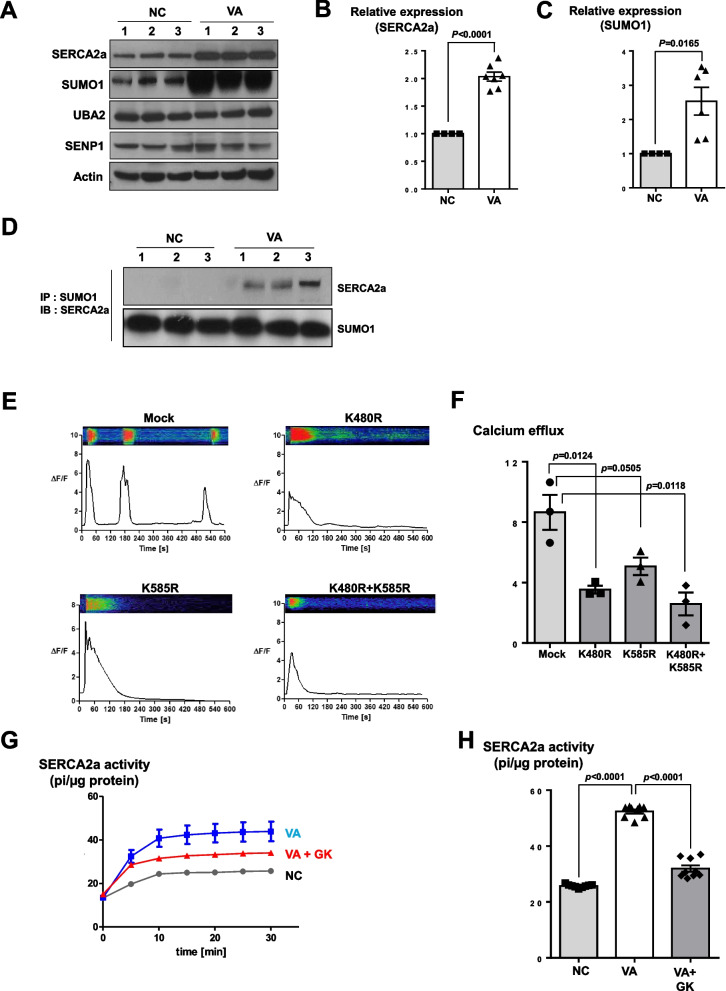
The enhanced SERCA2a activity of iPSC-derived VSMCs from VSA patient was induced by the increased small ubiquitin-related modifier (SUMO)ylation of SERCA2a. (**A-C**) Western blot shows that the amount of endogenous SERCA2a and SUMO1 proteins was greater in VSA patient-derived VSMCs than in normal subject-derived VSMCs (*n* = 4–7 per group). 50 μg of whole protein lysates from each sample were loaded with a 10% SDS-PAGE gel, transferred to nitrocellulose membranes, and probed with anti-SERCA2a (1:1000), anti-SUMO1 (1:1000), anti-UBA2 (1:1000), anti-SENP1 (1:1000), and anti-sarcomeric actin antibodies (1:1000). SUMO1 = Small Ubiquitin Like Modifier 1; UBA2 = Ubiquitin-like modifier-activating enzyme 2; SENP1 = Sentrin-specific protease 1. (**D**) Immunoprecipitation assay for SUMO1 and SERCA2a indicates that SUMO1 bound only to SERCA2a of VSMCs from VSA patients, not from normal subjects. (**E, F**) We transfected VSA-patient derived VSMCs with lentiviral vectors encoding SERCA2a with mutated lysine 480 and 585 residues. Each group was treated with Fluo-4 and monitored through time-lapse confocal microscopy over 10 min after the addition of 250 μM carbachol. The transfection with the mutant forms exhibited lower intracellular calcium efflux, indicating that these SUMOylated sites are responsible for the increased activity of SECAR2a in the VSMCs from VSA patients (*n* = 3 per group). (**G, H**) 10 μg of each cell lysate was mixed with the reaction buffer and treated with 1 mM ATP and Ginkgolic acid (10 μM), an inhibitor of SUMOylated E1 molecules. Ginkgolic acid suppressed enhanced SERCA2a activity in iPSC-derived VSMCs from VSA patients (*n* = 9 per group). *GK* Ginkgolic acid

**Fig. 5:**
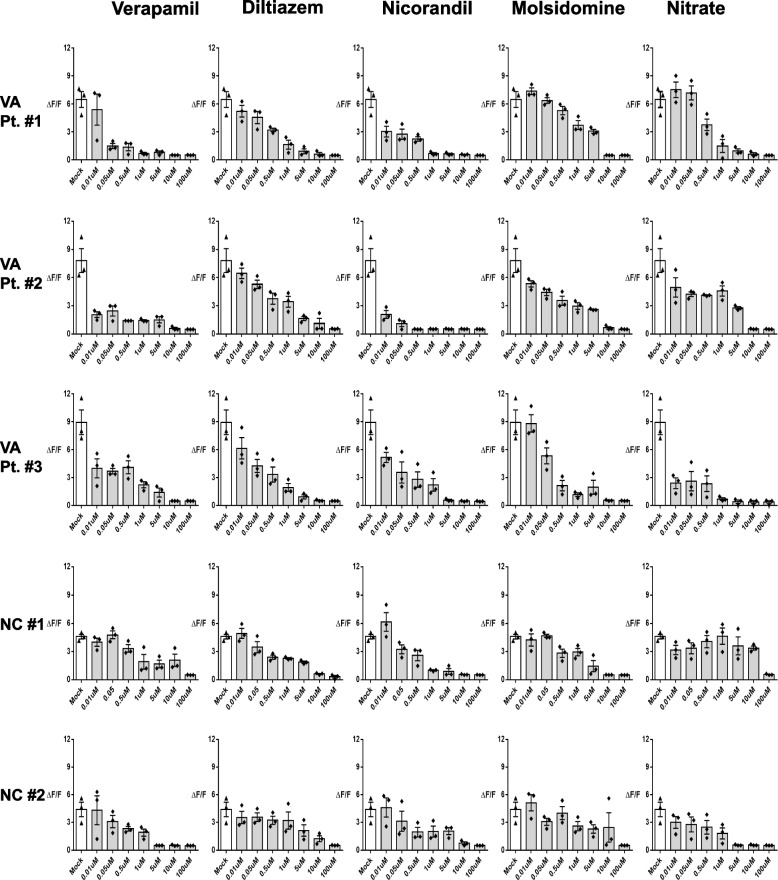
Differences in the magnitude of intracellular calcium efflux among VSA patients in response to various dosages of diverse vasodilators. By measuring intracellular calcium efflux in iPSC-derived VSMCs from VSA patients or normal subjects via our method, we evaluated changes of intracellular calcium efflux in response to various dosages (0.01, 0.05, 0.5, 1, 5, 10, 100 μM) of calcium channel blockers (Verapamil, Diltiazem) and vasodilators (Nicorandil, Molsidomine, Nitrate). The inhibitory effect of each drug differed even among VSA patients

### Increased level of sarco/endoplasmic reticulum Ca^2+^-ATPase (SERCA)2a activity and small ubiquitin-related modifier (SUMO)ylation in VSMCs derived from VSA patients

Calcium handling in sarco/endoplasmic reticulum (SR) may be the most important process underlying coronary artery spasm, because the SR is the key regulator of intracellular calcium. Therefore, we screened the activity of several calcium transporters in the SR, such as SERCA2a, (a calcium re-uptake regulator during excitation–contraction), and phospholamban (PLB, a key regulator of SERCA2a [20, 21]), and calcium release channels in the SR, such as ryanodine receptors (RYR), and inositol trisphosphate receptors (IP3R) [19, 22]. Among these, the protein levels of SERCA2a were elevated in VSA patient-derived VSMCs (Supplemental Fig. 6A). Both the total protein level and immunofluorescence intensity of SERCA2a in VSA group cells were higher than those in the normal group (Fig. 3A-3D). Moreover, the calcium-dependent ATPase activity assay indicated that SERCA2a activity in VSA group cells was greater than that in normal cells (Fig. 3E and 3F). After knocking down the expression of SERCA2a in VSA group cells with a lentiviral vector, high intracellular calcium efflux was restored to normal levels (Fig. 3G and 3H). In contrast, lentivirus-mediated overexpression of SERCA2a in control VSMCs enhanced intracellular calcium efflux, generating a second peak of calcium efflux (Fig. 3I and 3J). To investigate how SERCA2a protein was upregulated in the VSA group without changes in SERCA2a mRNA levels (Supplemental Fig. 6B-6D). Previous reports claim that SERCA2a activity can be modulated by post-translational modification (PTM) and that SERCA2a binds to small ubiquitin-related modifier1 (SUMO1) [23, 24]. Therefore, we examined the PTM of SERCA2a, with a focus on its SUMOylation. Only SUMO1 protein among ubiquitin-like modifier activating enzyme 2 (UBA2) and SUMO Specific peptidase 1 (SENP1) showed significantly higher levels in the VSA group cells than in the control group (Fig. 4A-4C, Supplemental Fig. 7A-7B). Furthermore, SUMO1 only bound to VSA group-derived SERCA2a (Fig. 4D). Therefore, SERCA2a PTM is likely to play an important role in VSA.

**Fig. 6:**
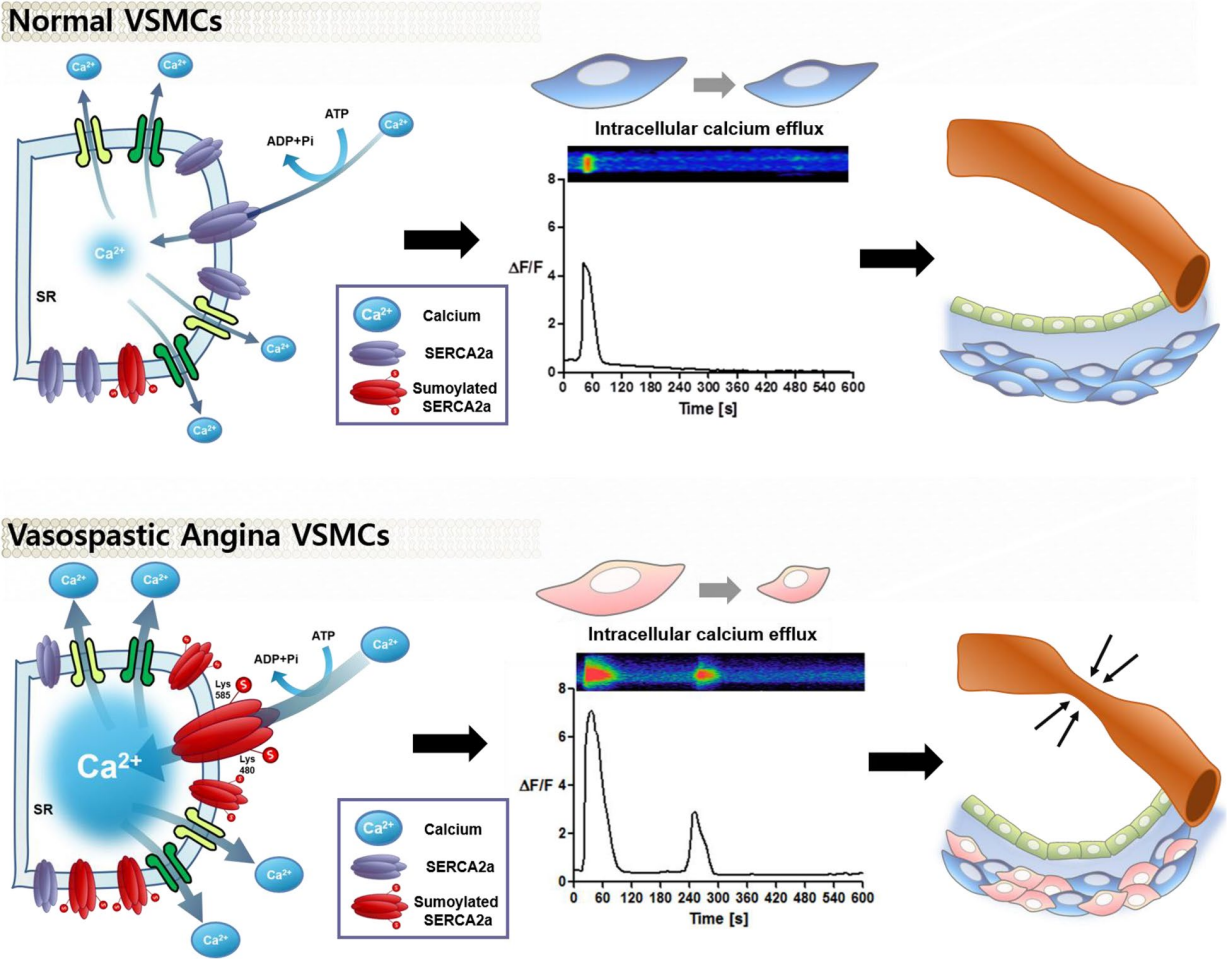
Schematic figure of the mechanism underlying VSMCs hyper-responsiveness in VSA patients. Compared to the status in normal subjects, the increased SUMOylation of SERCA2a in VSA patients enhances its stability, amount, and activity, in turn increasing intracellular calcium efflux and VSMCs hyperreactivity leading to vessel contraction

According to a previous report, SERCA2a is SUMOylated at lysines 480 and 585 [23]. We confirmed that the SUMOylated sites at lysines 480 and 585 were responsible for the increased calcium activity of SECAR2a in the VSA group using lentiviral vectors for the expression of mutant forms of the protein at lysine 480 and 585 (Fig. 4E and 4F). These results, imply that preventing SERCA2a SUMOylation in VSA patients may reduce hyperreactivity of VSMCs. Interestingly, treatment with ginkgolic acid [25], an inhibitor of SUMOlyated E1 molecules, reversed the increase of SERCA2a activity in the VSA group (Fig. 4G and 4H). These results suggest that the SUMOylation of SERCA2a may be a key factor in inducing hyperreactivity in VSMCs VSA patients.

### Different changes in intracellular calcium efflux in response to diverse vasodilators

Using our approach for the measurement of intracellular calcium efflux in VSA patient-specific iPSC-derived VSMCs, we evaluated changes in intracellular calcium efflux in response to vasodilators and calcium channel blockers (Fig. 5). The inhibitory effect of each drug differed even among the VSA patients as well as between VSA and normal groups. For example, the graphs of calcium efflux indicated that nicorandil was more effective than nitrate in patient #2, while nitrate was more effective than nicorandil in patient #3.

## Discussion

The current study revealed that the hyper-responsiveness of VSMCs from VSA patients may be triggered by the abnormal increase in intracellular calcium efflux, as compared to the control cells from subjects with negative provocation test results (Changes in the relative fluorescence unit [ΔF/F]; Control group vs. VSA group, 2.89 ± 0.34 vs. 10.32 ± 0.51, *p* < 0.01). Furthermore, this altered intracellular calcium regulation was caused by an increase in SERCA2a activity (pi/µg protein; control group vs. VSA group, vs. 25.75 ± 0.2 vs. 52.36 ± 0.71, *p* < 0.01), resulting from its increased SUMOylation (Fig. 6). A clear separation of the VSA and control group was observed around an ΔF/F value of 6 or 7, suggesting that this cut-off value could be utilized for biological diagnosis of VSA. In addition, the presence of a secondary calcium peak could be another diagnostic criterion for VSA.

Various studies have reported that myocardial infarction and life-threatening arrhythmias occur in approximately 25% of untreated or inadequately-treated patients with VSA [26, 27]. This highlights the need for an appropriate suspect and prompt diagnosis for VSA. Ethnic differences in the incidence of coronary artery spasm have been reported between Asians and Caucasians [28, 29]. Thus, underdiagnoses of less typical cases may occur in Western countries (Supplemental Figs. 8A-8C). Further, as the proportion of Asians in Western countries has recently increased, there is a possibility of higher VSA prevalence than expected in the Western world. Currently, the gold standard for VSA diagnosis is coronary angiography with a spasm provocation test. Although this approach seems safe compared to percutaneous coronary intervention, the reality is not so safe. The complication rate of the invasive provocation test was reported to be up to 4% including reperfusion ventricular fibrillation [30]. Considering this complication rate and the invasiveness of the provocation test, our ex vivo biological diagnostic method seems relatively safe and practical, using 10 mL of peripheral blood obtained in the outpatient department.

Pharmacological therapy is the main treatment strategy for VSA. Various drugs are prescribed, including nitrates, calcium channel blockers, nicorandil, molsidomine, and Rho-kinase inhibitors [26]. However, most VSA patient taking these medications suffer from side effects such as headache, leading to the discontinuation of drug treatment. Considering the risk of fatal complications in untreated VSA, drug compliance is of major importance. Therefore, personalized drug treatment strategies are necessary. However, no methods had been utilized for determining the appropriate drug for each patient until the introduction of iPSC models. In the current work with iPSCs-utilized disease modeling, we assessed which drugs were effective in suppressing the calcium response, and were able to choose fewer efficacious drugs at lower doses, resulting in lesser side effects.

Currently, VSMCs hyperreactivity and ECs dysfunction are considered the major abnormalities underlying coronary artery spasms [12, 13]. Our study demonstrated that the hyperreactivity of VSMCs in patients with VSA could be triggered by abnormal calcium efflux. We also evaluated the function of ECs in each group. We could not see any functional difference of iPSC-derived ECs between VSA patients and control subjects (Supplemental Figs. 3G and 3H). We speculated that iPSC-derived ECs might reflect the neonatal status of ECs. Thus, we examined another set of ECs that were differentiated directly from CiMS cells, because those ECs were representative of the current status of VSA patient ECs (biological aging process in adulthood). To our surprise, CiMS-derived ECs (adult stage) from VSA patients showed dysfunction compared to iPSC-derived ECs (neonatal stage) from the same patients (Supplemental Figs. 9A-9C). From these findings, we can infer the role of ECs on the natural course of coronary artery spasm. The NO produced by ECs at a young age is sufficient to suppress VSMCs hyperreactivity even in VSA patients. As the patient becomes older, the function of ECs decreases, compromising the protective and vasodilating effect of NO, which in turn leads to coronary artery spasm (Supplemental Fig. 10).

VSMCs hyperreactivity is triggered by diverse stimuli that act through various receptors and cellular mechanisms, complicating diagnosis and treatment [31, 32]. However, VSMCs contraction is finally mediated by the activation of actin-myosin filaments through increased intracellular calcium or enhanced calcium sensitivity [33]. The difference between VSA patient and normal subject in terms of the intracellular calcium activity was quite significant (Changes in the relative fluorescence unit [ΔF/F]; Control group vs. VSA group, 2.89 ± 0.34 vs. 10.32 ± 0.51). Moreover, we demonstrated a clear difference in intracellular calcium efflux between the VSA group and the control group around an ΔF/F value of 6 or 7 (change in the relative fluorescence unit), suggesting that this cut-off value could be used for ex vivo diagnosis of VSA. In addition, the presence or absence of a secondary calcium peak could be utilized as another diagnostic criteria for VSA.

Our findings revealed that the increased SERCA2a expression in VSA patients is caused by enhanced SUMOylation of the SERCA2a protein, protecting it against degradation (Central Illustration). Therefore, it suggests that inhibiting SUMOylation of SERCA2a in VSA patients may suppress coronary artery spasm. There have been relatively few studies on SUMOylation inhibitors [23]. We observed that ginkgolic acid could suppress the activity of SERCA2a, inhibiting VSMCs hyper-responsiveness. One systemic review of 23 randomized clinical studies with a total sample size of 2,529 participants revealed that ginkgo biloba treatment had beneficial effects in patients with angina pectoris [34]. Taken together, these findings suggest that the pharmacological modulation of SERCA2a SUMOylation could represent a promising strategy for the control of coronary artery spasm. In addition, our findings could be applied to other diseases such as bronchial asthma. Since the hyper-responsiveness of bronchial smooth muscle cells is known to be one of the well-known causes of bronchial asthma, the findings of our study could be utilized as another diagnostic criteria for bronchial asthma after differentiating bronchial smooth muscle cells from iPSCs.

Our study has several limitations. Although our study showed the alteration of calcium handing in SR in VSMCs in in vitro, we could not prove the direct causal relationship between this alteration and coronary artery spasm in in vivo models. If we could make the vessel structure using iPSC-derived cells, this strategy could provide direct evidence for the pathophysigoilogy of VSA. In addition, this study included the limited number of subjects in terms of the analysis of VSA characteristics. Therfore, further study including large number of patients is required to validate the clinical implications of our study.

## Conclusion

Our novel ex vivo biologic approach utilizing peripheral blood demonstrated that the different response in intracellular calcium efflux of VSMCs could represent a diagnostic marker for VSA. Moreover, our findings highlight that calcium handling in the SR could underpin coronary artery spasm. Taken together, the novel method described herein could be a useful tool for further research on unknown pathobiology, diagnosis, and drug development for VSA.

## Supplementary Information

**Additional file 1:** **Supplemental video 1.**

**Additional file 2:** **Supplemental video 2.**

**Additional file 3. Supplemental data.**

## Data Availability

Please, contact the corresponding author for data requests.

## References

[1] Yasue H, Kugiyama K (1997). Coronary spasm: clinical features and pathogenesis. Intern Med.

[2] Nakamura M, Takeshita A, Nose Y (1987). Clinical characteristics associated with myocardial infarction, arrhythmias, and sudden death in patients with vasospastic angina. Circulation.

[3] Myerburg RJ, Kessler KM, Mallon SM, Cox MM, deMarchena E, Interian A (1992). Life-Threatening Ventricular Arrhythmias in Patients with Silent Myocardial Ischemia Due to Coronary Artery Spasm. N Engl J Med.

[4] Saleh TA (2021). Protocols for synthesis of nanomaterials, polymers, and green materials as adsorbents for water treatment technologies. Environ Technol Innov.

[5] Saleh TA (2020). Nanomaterials: Classification, properties, and environmental toxicities. Environ Technol Innov.

[6] Lip GYH, Ray KK, Shiu MF (1998). Coronary spasm in acute myocardial infarction. Heart.

[7] Bory M, Pierron F, Panagides D, Bonnet JL, Yvorra S, Desfossez L (1996). Coronary artery spasm in patients with normal or near normal coronary arteries. Long-term follow-up of 277 patients. Eur Heart J..

[8] Walling A, Waters DD, Miller DD, Roy D, Pelletier GB, Theroux P (1987). Long-term prognosis of patients with variant angina. Circulation.

[9] Waters DD, Miller DD, Szlachcic J, Bouchard A, Méthé M, Kreeft J (1983). Factors influencing the long-term prognosis of treated patients with variant angina. Circulation..

[10] Severi S, Daves G, Maseri A, Marzullo P, L’abbate A (1980). Long-term prognosis of “variant” angina with medical treatment. Am J Cardiol.

[11] Ahn JM, Lee KH, Yoo SY, Cho YR, Suh J, Shin ES (2016). Prognosis of Variant Angina Manifesting as Aborted Sudden Cardiac Death. J Am Coll Cardiol.

[12] Kaski JC, Crea F, Meran D, Rodriguez L, Araujo L, Chierchia S (1986). Local coronary supersensitivity to diverse vasoconstrictive stimuli in patients with variant angina. Circulation.

[13] Vanhoutte PM, Shimokawa H (1989). Endothelium-derived relaxing factor and coronary vasospasm. Circulation.

[14] Yang HM, Kim JY, Cho HJ, Lee JE, Jin S, Hur J (2020). NFATc1+CD31+CD45- circulating multipotent stem cells derived from human endocardium and their therapeutic potential. Biomaterials..

[15] Takahashi K, Tanabe K, Ohnuki M, Narita M, Ichisaka T, Tomoda K (2007). Induction of pluripotent stem cells from adult human fibroblasts by defined factors. Cell.

[16] Lipskaia L, Keuylian Z, Blirando K, Mougenot N, Jacquet A, Rouxel C (2014). Expression of Sarco (Endo) plasmic Reticulum Calcium ATPase (SERCA) system in normal mouse cardiovascular tissues, heart failure and atherosclerosis. Biochim Biophys Acta.

[17] Nelson MT, Cheng H, Rubart M, Santana LF, Bonev AD, Knot HJ (1995). Relaxation of arterial smooth muscle by calcium sparks. Science.

[18] Jaggar JH, Porter VA, Jonathan Lederer W, Nelson MT (2000). Calcium sparks in smooth muscle. Am J Physiol Cell Physiol..

[19] Hill-Eubanks DC, Werner ME, Heppner TJ, Nelson MT (2011). Calcium signaling in smooth muscle. Cold Spring Harb Perspect Biol.

[20] MacLennan DH, Kranias EG (2003). Phospholamban: a crucial regulator of cardiac contractility. Nat Rev Mol Cell Biol.

[21] Wray S, Burdyga T (2010). Sarcoplasmic reticulum function in smooth muscle. Physiol Rev.

[22] Neylon CB, Richards SM, Larsen MA, Agrotis A, Bobik A (1995). Multiple types of ryanodine receptor/Ca2+ release channels are expressed in vascular smooth muscle. Biochem Biophys Res Commun.

[23] Kho C, Lee A, Jeong D, Oh JG, Chaanine AH, Kizana E (2011). SUMO1-dependent modulation of SERCA2a in heart failure. Nature.

[24] Meyer M, Schillinger W, Pieske B, Holubarsch C, Heilmann C, Posival H (1995). Alterations of sarcoplasmic reticulum proteins in failing human dilated cardiomyopathy. Circulation.

[25] Fukuda I, Ito A, Hirai G, Nishimura S, Kawasaki H, Saitoh H (2009). Ginkgolic acid inhibits protein SUMOylation by blocking formation of the E1-SUMO intermediate. Chem Biol.

[26] JCS Joint working group (2014). Guidelines for diagnosis and treatment of patients with vasospastic angina (Coronary Spastic Angina) (JCS 2013). Circ J.

[27] Kishida H, Tada Y, Tetsuoh Y, Yamazaki Y, Saito T, Fukuma N (1991). A new strategy for the reduction of acute myocardial infarction in variant angina. Am Heart J.

[28] Takagi Y, Yasuda S, Tsunoda R, Ogata Y, Seki A, Sumiyoshi T (2011). Clinical characteristics and long-term prognosis of vasospastic angina patients who survived out-of-hospital cardiac arrest: multicenter registry study of the Japanese Coronary Spasm Association. Circ Arrhythm Electrophysiol.

[29] Pristipino C, Beltrame JF, Finocchiaro ML, Hattori R, Fujita M, Mongiardo R (2000). Major racial differences in coronary constrictor response between japanese and caucasians with recent myocardial infarction. Circulation.

[30] Sueda S, Kohno H (2016). Overview of complications during pharmacological spasm provocation tests. J Cardiol.

[31] Crea F, Chierchia S, Kaski JC, Davies GJ, Margonato A, Miran DO (1986). Provocation of coronary spasm by dopamine in patients with active variant angina pectoris. Circulation.

[32] McFadden EP, Clarke JG, Davies GJ, Kaski JC, Haider AW, Maseri A (1991). Effect of intracoronary serotonin on coronary vessels in patients with stable angina and patients with variant angina. N Engl J Med.

[33] Hirano K (2007). Current topics in the regulatory mechanism underlying the Ca2+ sensitization of the contractile apparatus in vascular smooth muscle. J Pharmacol Sci.

[34] Sun T, Wang X, Xu H (2015). Ginkgo Biloba extract for angina pectoris: a systematic review. Chin J Integr Med.

