# Supplementary material for: Identification of cell-biologic mechanisms of coronary artery spasm and its ex vivo diagnosis using peripheral blood-derived iPSCs
Source: Biomater Res. 2023 Feb 18;27:16. doi: 10.1186/s40824-023-00345-2 (PMC9938986; doi:10.1186/s40824-023-00345-2)
Supplement: Supplementary file 3 — Additional file 3. Supplemental data. [file 40824_2023_345_MOESM3_ESM.docx]

**Online Supplementary Data**

**Supplemental Tables 1 and 2**

**Supplemental Figure Legends**

**References**

**Supplemental Figures 1-10**

**Supplemental Table**

**Supplemental Table 1. Baseline characteristics of the patients**


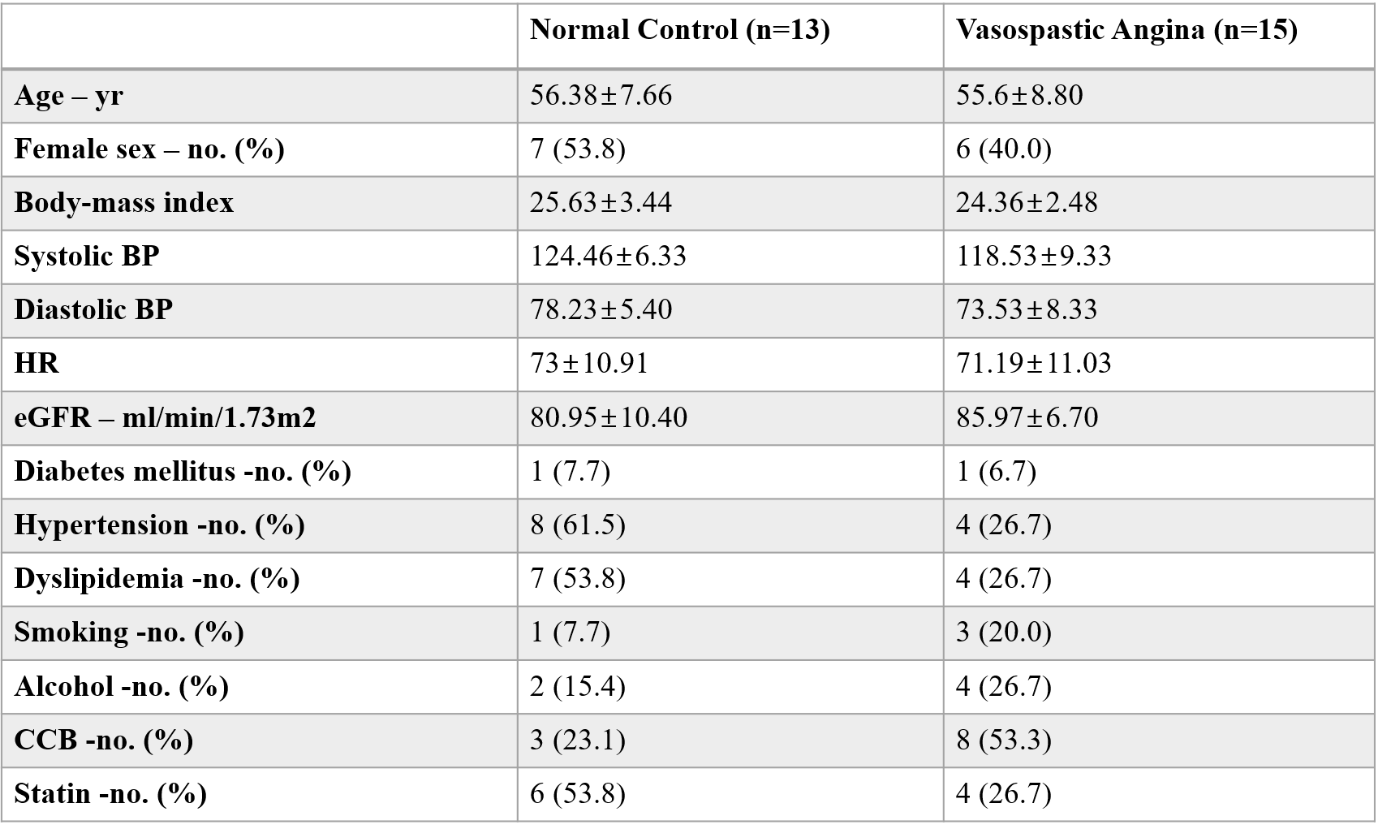


**Supplemental Table 2. List of primers**

| **Gene** | **Primer sequence (5’ -> 3’)** |
| --- | --- |
| SERCA2a | F: TTTGAAGGCGTGGATTGTG |
|  | R: CTGGAAGATGAGTGGCAAGG |
| PLB | F: ATGGAGAAAGTCCAATACCT |
|  | R: AGAAGCATCACGATGATACA |
| RYR2 | F: GACCGAACCAAGAAATCCAA |
|  | R: ACAACCAGGACGACTCCAAC |
| IP3R1 | F: GTGACAGGAAACATGCAGACTCG |
|  | R: CAGCAGTTGCACAAAGACAGGC |
| NANOG | F: AGACTGTCTCTCCTCTTCCT |
|  | R: GGCTGAGGTATTTCTGTCTC |
| OCT3/4 | F: GACAGGGGGAGGGGAGGAGC |
|  | R: CTTCCCTCCAACCAGTTGCC |
| Brachyury T | F: TGCTTCCCTGAGACCCAGTT |
|  | R: GATCACTTCTTTCCTTTGCA |
| CNN1 | F: GTCCACCCTCCTGGCTTT |
|  | R: AAACTTGTTGGTGCCCATCT |
| SMA | F: CACTGTCAGGAATCCTGTGA |
|  | R: CAAAGCCGGCCTTACAGA |
| SMA22a | F: GCCTTCTTTCCCCAGACA |
|  | R: CCTCCAGCTCCTCGTCAT |
| PECAM | F: AAGTGGAGTCCAGCCGCATATC |
|  | R: ATGGAGCAGGACAGGTTCAGTC |
| VeCAD | F: CCTCACTTCCCCATCATGTA |
|  | R: CCAGCCTCTCAATGGCGAAC |
| GAPDH | F: GTCTCCTCTGACTTCAACAGCG |
|  | R: ACCACCCTGTTGCTGTAGCCAA |
| 18s | F: CCTGCGGCTTAATTTGACTC |
|  | R: ACCAACTAAGAAGAACGGCCATG |
| Actin | F: CACCATTGGCAATGAGCGGTTC |
|  | R: AGGTCTTTGCGGATGTCCACGT |

**Supplemental Figure Legends**

**Supplemental Figure 1. Generation and characterization of human induced pluripotent stem cells (iPSCs) from Circulating Mulipotent Stem (CiMS) cells of normal subjects or vasospastic angina** **(VSA) patients**

(A) CiMS cells are ‘circulating multipotent stem’ cells in human peripheral blood and are originated from human endocardium, which was discovered in our previous research [1]. They are stem cells having potential to differentiate into multiple lineages and thus easily reprogrammed to iPSCs. We established protocol to culture CiMS cells from human peripheral blood and to reprogram them into iPSCs. Morphologies on phase contrast images of CiMS cells and iPSCs from normal subjects and VSA patients (scale bar, 400 µm). NC = negative control group with a negative provocation test; VA = vasospastic angina group with a positive provocation test. (B) Alkaline phosphatase-positive staining (scale bar, 400 µm) and immunofluorescence labeling with stemness markers such as NANOG and OCT3/4, showed high expression levels in normal NC- and VSA patient-derived iPSCs (scale bar, 50 µm). (C, D) The expression levels of pluripotency markers, such as OCT3/4 and NANOG, were determined for NC- and VSA patient-derived iPSCs via qRT-PCR (n=3-9 per group; hereafter, n represents the number of biological replicates). hES = human embryonic stem cells. (E) Verification of three-germ-layer markers in differentiated pluripotent stem cells. Ectodermal lineage differentiation of iPSCs were simultaneously stained with anti-OTX2 (1:100), mesodermal lineage differentiation of iPSCs were stained with anti-Brachyury T (1:100), and endodermal lineage differentiation of iPSCs were stained with anti-SOX17 (1:100). All nuclei were counterstained with 4′, 6-diamidino-2-phenylindole (DAPI) (scale bar, 50 µm). OTX = Orthodenticle Homeobox; T= Brachyury T; SOX17 = SRY-Box Transcription Factor 17. (F) Karyotype analysis of iPSCs from normal control and VSA patients. (G). Hematoxylin and Eosin (H&E) staining representing all three germ layers (ectoderm, mesoderm, and endoderm) in the teratoma that was generated in the severe combined immunodeficient (SCID) mouse (scale bar, 100 µm). All these findings suggest that iPSCs in our study were well generated and healthy.

**Supplemental Figure 2. Differentiation and characterization of human vascular smooth muscle cells (VSMCs) from iPSCs of normal subjects and VSA patients**

(A) Schematic representation of single cell-based human VSMCs differentiation protocols from iPSCs of normal subjects or VSA patients. (B, C) Flow cytometry analysis and quantification of T expression levels in single cell iPSCs treated with CHIR99021 (10 µM) for 2 days (n=4 per group). (D) The gene expression levels of *T* were determined via qRT-PCR in single cell iPSCs treated with CHIR99021 (10 µM) for 2 days (n=3-9 per group). (E) Phase contrast images (scale bar, 400 µm) and immunofluorescence CNN1 (1:100) and SMA (1:100) of differentiated VSMCs (scale bar, 50 µm). BF indicates bright field. CNN1 = calponin 1; SMA = smooth muscle actin (F) Western blot analysis representing the expression levels of endogenous CNN1, SM22α, and SMA in the iPSC-derived VSMCs of normal control and VSA patients (N.S: not significant; NC vs. VA; n=3). 10 μg of whole protein lysates from each sample were loaded with a 10% SDS-PAGE gel, transferred to nitrocellulose membranes and probed with anti-CNN1 (1:1000), anti- SM22α (1:1000), anti- SMA (1:1000), and anti-sarcomeric actin antibodies (1:1000). SM22α = smooth muscle protein 22 alpha (G-I) The gene expression levels of *CNN1*, *SMA*, and *SM22α* were determined via qRT-PCR in the iPSC-derived VSMCs of normal control and VSA patients (n=3-9 per group).

**Supplemental Figure 3. Differentiation and characterization of human endothelial cells (ECs) from iPSCs of normal subjects and VSA patients**

(A) Schematic representation of single cell-based ECs differentiation protocols from iPSCs of normal control and VSA patient. (B, C) The gene expression levels of *PECAM* and *VeCAD* were determined via qRT-PCR in iPSC-derived ECs from normal control and VSA patient (n=3 per group). PECAM = platelet endothelial cell adhesion molecule; VeCAD = vascular endothelial cadherin (D) Phase contrast images (scale bar, 400 µm), and immunofluorescence staining for PECAM and VeCAD of iPSC-derived ECs (scale bar, 50 µm). BF indicates bright field. (E) iPSC-derived ECs from normal control and VSA patient, 5 × 10^4^ cells were seeded on matrigel and the formation of vascular tube-like structures was quantified. Images were taken after 18 hours and analyzed using the Image J software (scale bar, 400 µm) (n=11 per group). (F) iPSC-derived ECs at day 17 were analyzed using the Acetylated –low density lipoprotein (Ac-LDL) uptake assay. LDL is shown in red (n=3 per group; scale bar, 10 µm). (G) Nitric oxide (NO) was detected with diaminofluorescein-2 diacetate (DAF-2DA, 1 µM) and tracked for 60 minutes. DAF-2DA intensity was quantified and represented as a bar graph (n=8 per group; scale bar, 50 µm). (H) The concentration of nitric oxide (NO) from iPSC-derived ECs from normal control were determined using the NO Detection Kit (n=8 per group).

**Supplemental Figure 4. Live imaging of changes in intracellular calcium efflux in iPSC-derived VSMCs from normal control and VSA patient**

(A) Still Images of calcium efflux using Fluo-4 obtained by laser-scanning confocal microscopy. Intracellular calcium efflux in response to carbachol (250 μM) treatment was measured with Fluo-4. The VSA group showed significantly higher intensity of calcium efflux than normal control group.

**Supplemental Figure 5. Live imaging and analysis of intracellular calcium efflux in iPSC-derived VSMC spheroids of normal subjects and VSA patients**

(A) Schematic representation of human VSMC spheroid generation protocols from iPSC-derived human VSMCs of normal control and VSA patient. (B, C) Still Images of calcium efflux using Fluo-4 obtained by laser-scanning confocal microscopy. Intracellular calcium efflux in response to carbachol (250 μM) treatment was measured with Fluo-4. The iPSC-derived VSMC spheroids from VSA patient exhibited significantly higher intensity and more peaks of calcium efflux than those from normal subjects.

**Supplemental Figure 6. Analysis of several calcium transporters in iPSC-derived VSMCs from normal subjects and VSA patients**

(A) Western blot analysis represented the expression levels of endogenous SERCA2a, tPLB, pPLB, tRYR, IP3R, and actin in iPSC-derived human VSMCs from normal control and VSA patients. Only SERCA2a protein level was increased in iPSC-derived human VSMCs of VSA patients compared with normal subjects. 10 μg of whole protein lysates from each sample were loaded with a 10% SDS-PAGE gel, transferred to nitrocellulose membranes and probed with anti-SERCA2a (1:1000), anti-tPLB (1:1000), anti-pPLB (1:1000), anti-tRYR (1:1000), anti-IP3R (1:1000), and anti-sarcomeric actin antibodies (1:1000). SERCA2a = [sarco](https://en.wikipedia.org/wiki/Sarcoplasmic_reticulum)/[endoplasmic reticulum](https://en.wikipedia.org/wiki/Endoplasmic_reticulum) [Ca](https://en.wikipedia.org/wiki/Calcium)^2+^-[ATPase](https://en.wikipedia.org/wiki/ATPase) 2a; tPLB = total phospholamban; pPLB = phosphorylated phospholamban; tRYR = total Ryanodine receptor; IP3R = inositol 1,4,5-trisphosphase (B) The gene expression levels of *SERCA2a*, *PLB*, *RYR*, *IP3R*, and *ACTIN* were determined using RT-PCR in iPSC-derived human VSMCs from normal control and VSA patients. No difference of each gene was observed between groups. (C, D) The endogenous gene expression level of SERCA2a was determined via RT-PCR and qRT-PCR in iPSC-derived human VSMCs from normal control and VSA patients (n=3 per group).

**Supplemental Figure 7. Quantitative comparison of SUMOylation-related proteins in iPSC-derived VSMCs between normal subjects and VSA patients**

(A) Relative expression levels of UBA2 (n=3 per group). (B) Relative expression levels of SENP1 (n=3-6 per group). UBA2 = Ubiquitin-like modifier-activating enzyme 2; SENP1 = Sentrin-specific protease 1.

**Supplemental Figure 8. A case report of vasospastic angina**

(A) A 72-year-old Asian woman who had undergone coronary artery bypass graft (CABG) in the United States visited our hospital owing to recurrent chest pain. Her coronary angiography demonstrated a focal tight stenosis at mid left anterior descending (LAD) coronary artery. Arrow indicates stenotic portion of LAD. (B) After intracoronary infusion of nitroglycerin, the vessels were fully dilated and the tight stenosis at mid LAD disappeared. Arrow indicates the previous stenotic portion of LAD. (C) The left internal thoracic artery anastomosed with LAD was regressed completely. We finally diagnosed the patient with VSA, and she was discharged with anti-spastic medications. Importantly, our ex vivo diagnostic method indicated a value of calcium efflux over the cut-off threshold. At follow-up 1 year later, the patient no longer complained of chest pain.

**Supplemental Figure 9. Difference in NO generation between iPSC-derived ECs (representing neonatal phase) and CiMS cell-derived ECs (representing adult phase) in VSA patients**

(A) Schematic protocol figure of ECs differentiated directly from CiMS cells of normal control and VSA patient. (B) The intensity of NO was detected with DAF-2DA (1 µM) in ECs differentiated directly from iPSCs or CiMS cells of VSA patients. NO intensity was higher in ECs from iPSCs than from CiMS cells (n=8 per group). (C) The concentration of NO was higher in ECs from iPSCs than from CiMS cells (n=9 per group). These data indicate that endothelial function becomes worse in aged subjects.

**Supplemental Figure 10. The proposed mechanism of the decreasing vasodilatory effect of ECs according to aging, leading to onset of VSA in the adulthood**

NO generated from ECs at a young age could be sufficient to suppress VSMCs hyperreactivity. When patients become older, the function of ECs decreases. This contributes to the reduced vasodilatory effect of NO on VSMCs, leading to coronary artery spasm.

**References**

1. Yang HM, Kim JY, Cho HJ, et al. NFATc1+CD31+CD45- circulating multipotent stem cells derived from human endocardium and their therapeutic potential. Biomaterials 2020;232:119674.
